# Supplementary material for: Scalable Generation of Nanovesicles from Human-Induced Pluripotent Stem Cells for Cardiac Repair
Source: Int J Mol Sci. 2022 Nov 18;23(22):14334. doi: 10.3390/ijms232214334 (PMC9696585; doi:10.3390/ijms232214334)
Supplement: Supplementary file 1 [file ijms-23-14334-s001.zip › Supplementary Figures Caption.pdf]

Supplementary Figure S1. iPSC model characterization. Florescence microscopy of iPSC models (CL2 and CERA) based on A) PI/Hoechst staining for cell viability and B) Ki67/Hoechst staining for cell proliferation assay. Scale bar, 250 μm

Supplementary Figure S2. Nanoparticle analysis of NVs following freeze-thaw cycles. Single particle tracking analysis of NVs following single (1) or repeated (5-10) free-thaw cycle(s) on dry-ice. Particle diameter was assessed by ZetaView (Particle Metrix, PMX-120) in experimental triplicate; 11 positions were captured with the following parameters: camera sensitivity: 80, min area: 5, max area: 1000, brightness: 30, min trace length: 15, temperature: 25°C. Calibration beads (Nano FCM, S16M-Exo) were used for instrument setup. Capture was performed at medium video setting, corresponding to 30 frames per position. ZetaView software 8.5.10 was used to analyse acquired data. For freeze-thaw analyses, NVs were placed on dry ice and this cycle repeated for 1, 5, and 10 -times before NTA analysis.

Supplementary Figure S3. Proteome analysis of iPSC and NVs. A) Venn Diagram of parental cells vs NVs proteome (CERA and CL2, respectively). B) EnrichmentMap analysis for iPSCs and NVs, differential analysis was performed (p<0.05, fold change (FC): ±1.5)) and components enriched in cells (blue) or NVs (orange) were analysed using Cytoscape and Enrichment map (cellular component). The node size corresponds to the number of proteins involved in that cellular component.

Supplementary Figure S4. NTA analysis of density gradient fractionation. Concentration of particles per mL plot on each fraction collected from the centrifuged gradient. Particle diameter distribution among the collected fractions. *p<0.05, **p<0.0052.

Supplementary Figure S5. Gene ontology analysis of non-encapsulated proteins. Proteins not encapsulated within NVs are primarily nuclear components (Cellular component) and nucleic acid binding (Molecular Function). Term size 5-500

Supplementary Figure S6. Confocal microscopy analysis of NV uptake to target cells. Confocal microscopy analysis along Z-axis (inset) reveal uptake of NVs to target cells. Image resolution Z-stack of approximately 15 μm, 25 steps. Resolution of 1024 × 1024, 8× averaging 2.4 dwell time, Objective 20X, Digital Zoom 20x. * Scale bar, 5 μm.

Supplementary Figure S7. Hierarchical cluster analysis of target cell proteome following NV treatments. Hierarchical cluster analysis of NV treatments CL2 or CERA) and vehicle controls for each assay were determined (ANOVA, P<0.05, fold change (FC): 1.5), to reveal distinct clusters of differential protein expression for each assay; A) cardiomyocyte (CM) survival, B) endothelial (HUVEC) tube formation, and C) TGF-β-mediated human primary cardiac fibroblast (hCF) activation. Comparative analysis of each NV treatment group relative to control treatments as shown (UT, untreated; hypoxia, vehicle treated hypoxia alone; TGF, TGF-β treatment alone).
