# Supplementary figures and images for: Scalable Generation of Nanovesicles from Human-Induced Pluripotent Stem Cells for Cardiac Repair
Source: Int J Mol Sci. 2022 Nov 18;23(22):14334. doi: 10.3390/ijms232214334 (PMC9696585; doi:10.3390/ijms232214334)

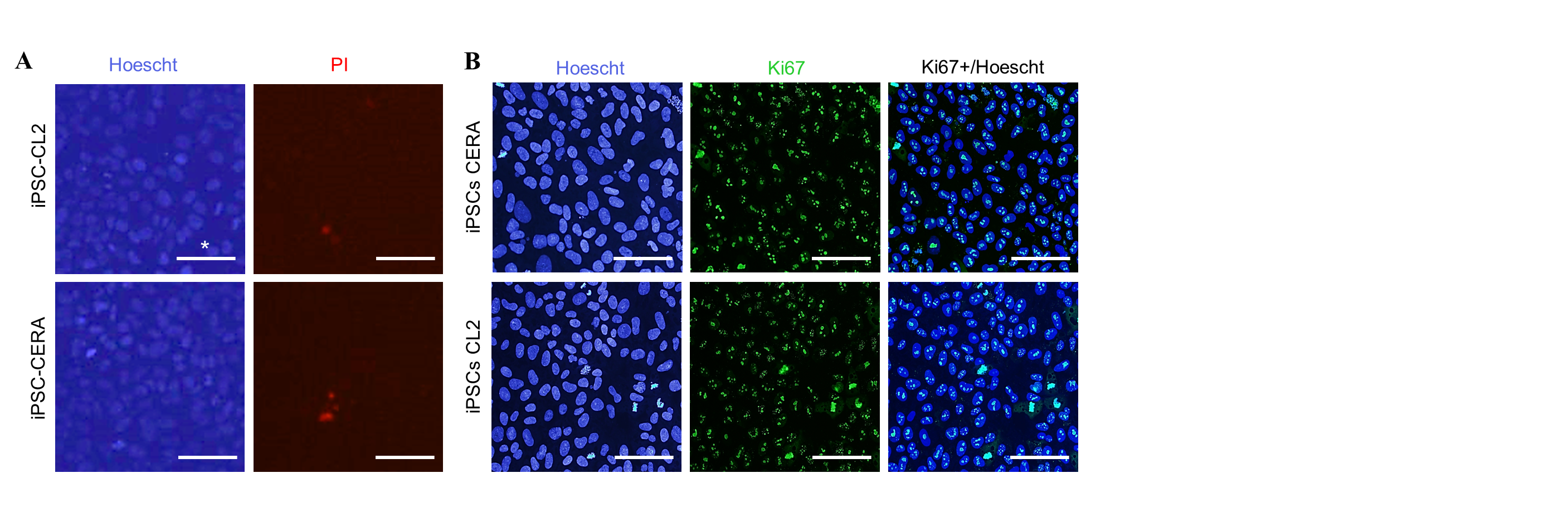

Supplement: Supplementary file 1 [file ijms-23-14334-s001.zip › Supplementary Figure S1.png]

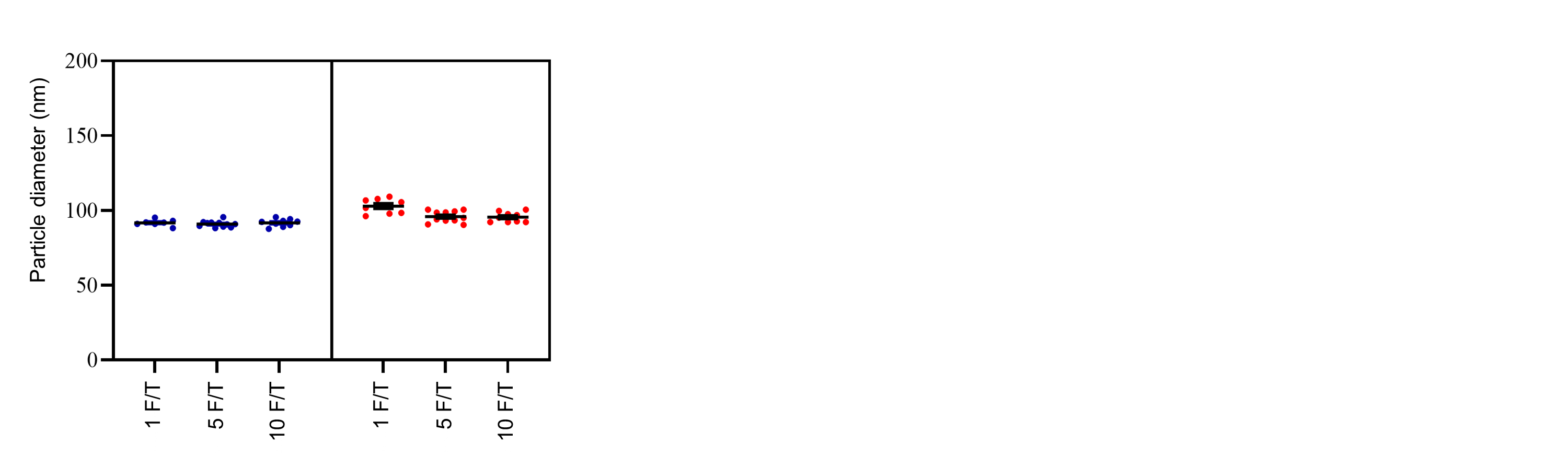

Supplement: Supplementary file 1 [file ijms-23-14334-s001.zip › Supplementary Figure S2.png]

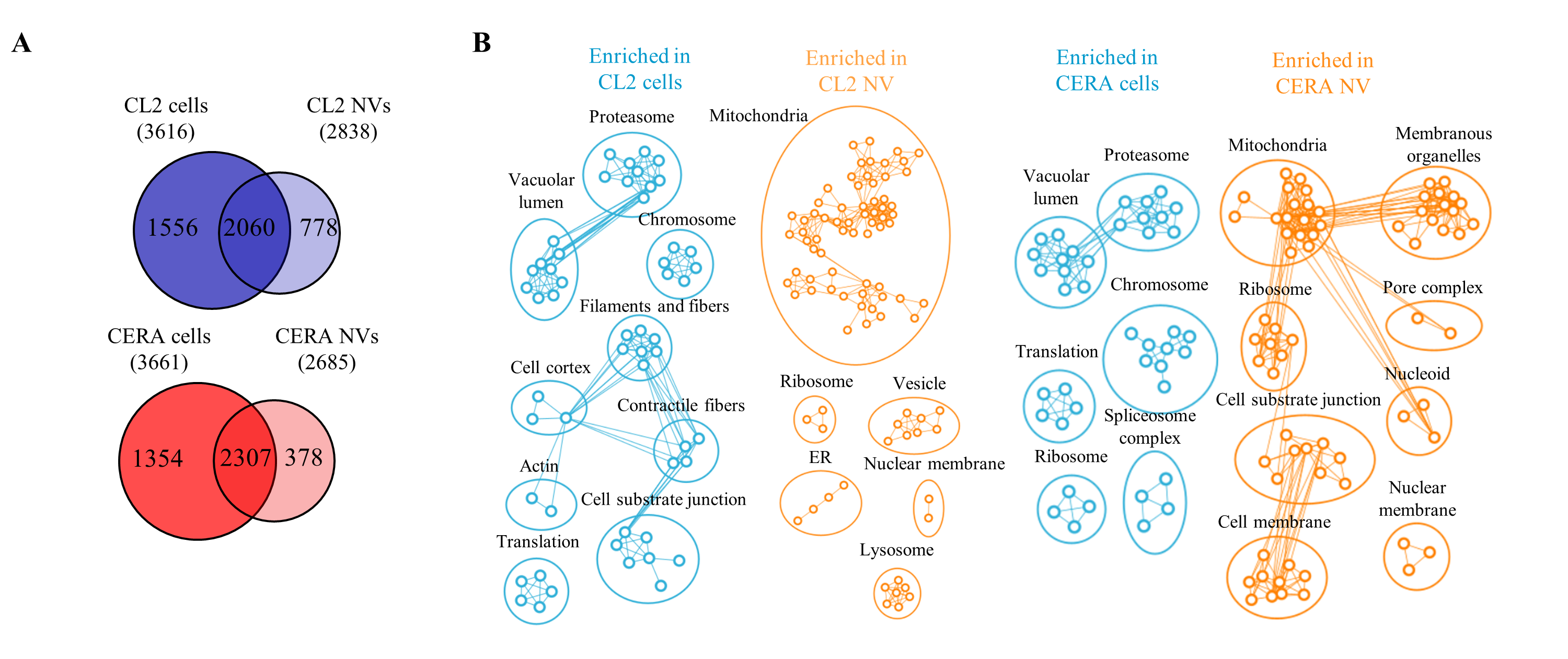

Supplement: Supplementary file 1 [file ijms-23-14334-s001.zip › Supplementary Figure S3.png]

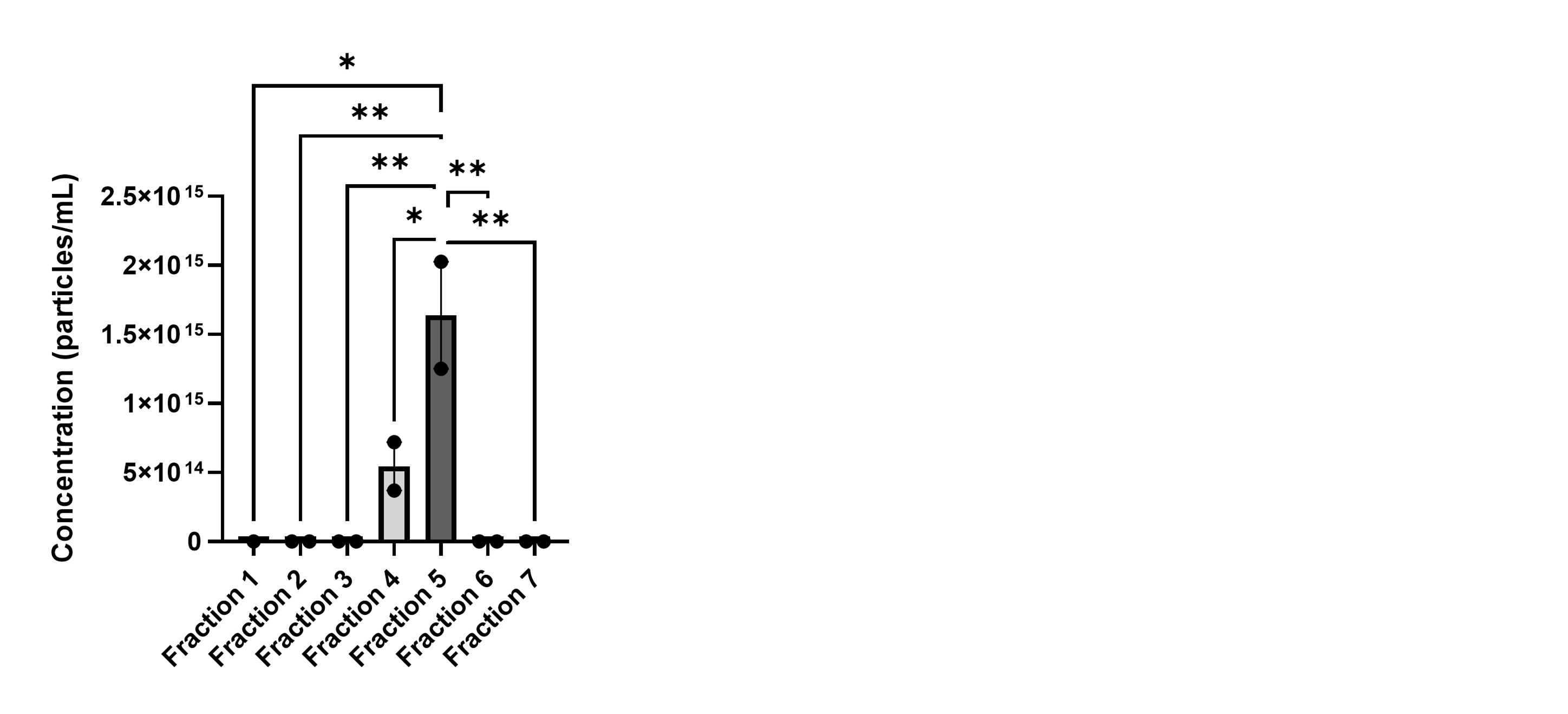

Supplement: Supplementary file 1 [file ijms-23-14334-s001.zip › Supplementary Figure S4.png]

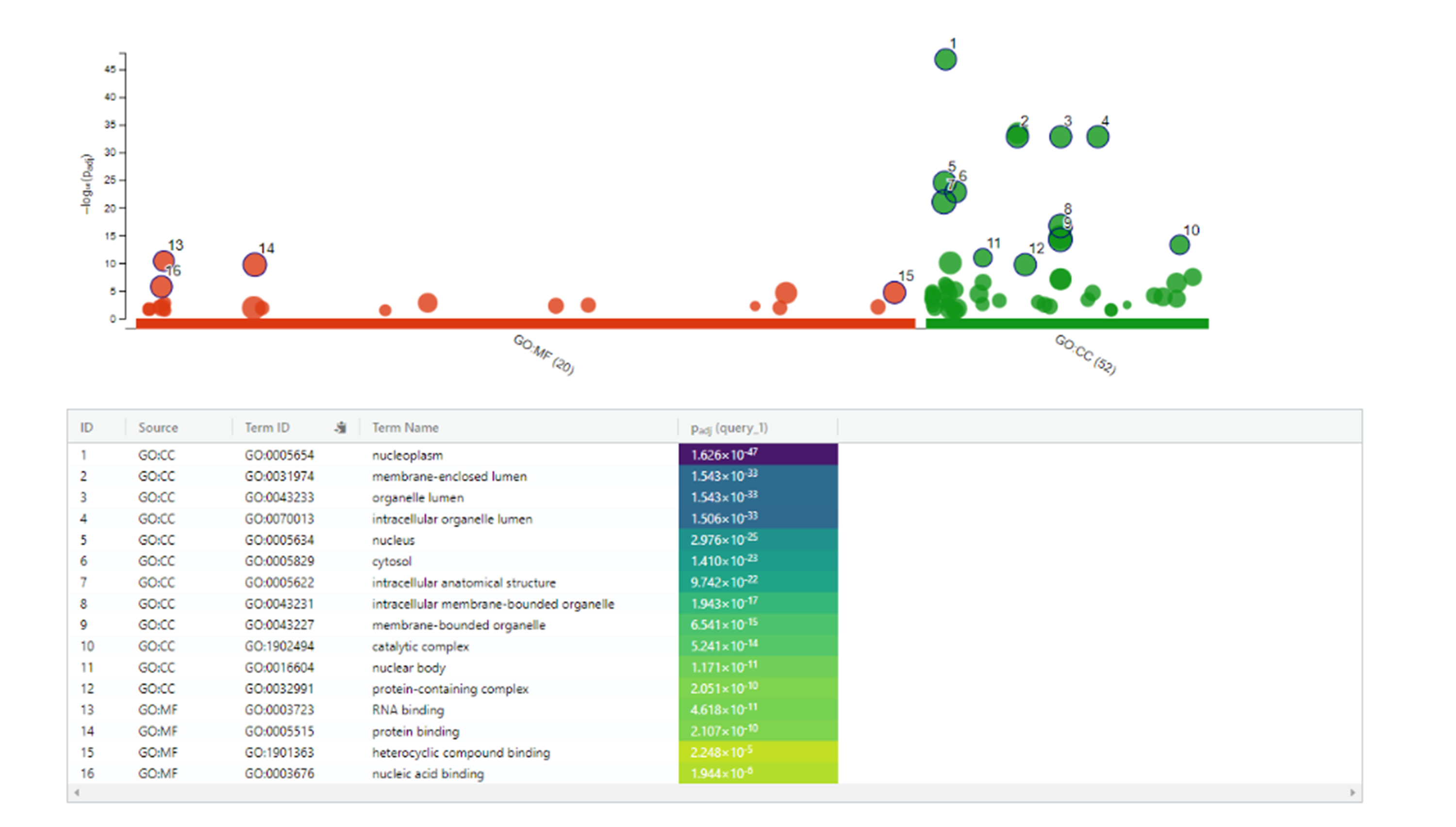

Supplement: Supplementary file 1 [file ijms-23-14334-s001.zip › Supplementary Figure S5.png]

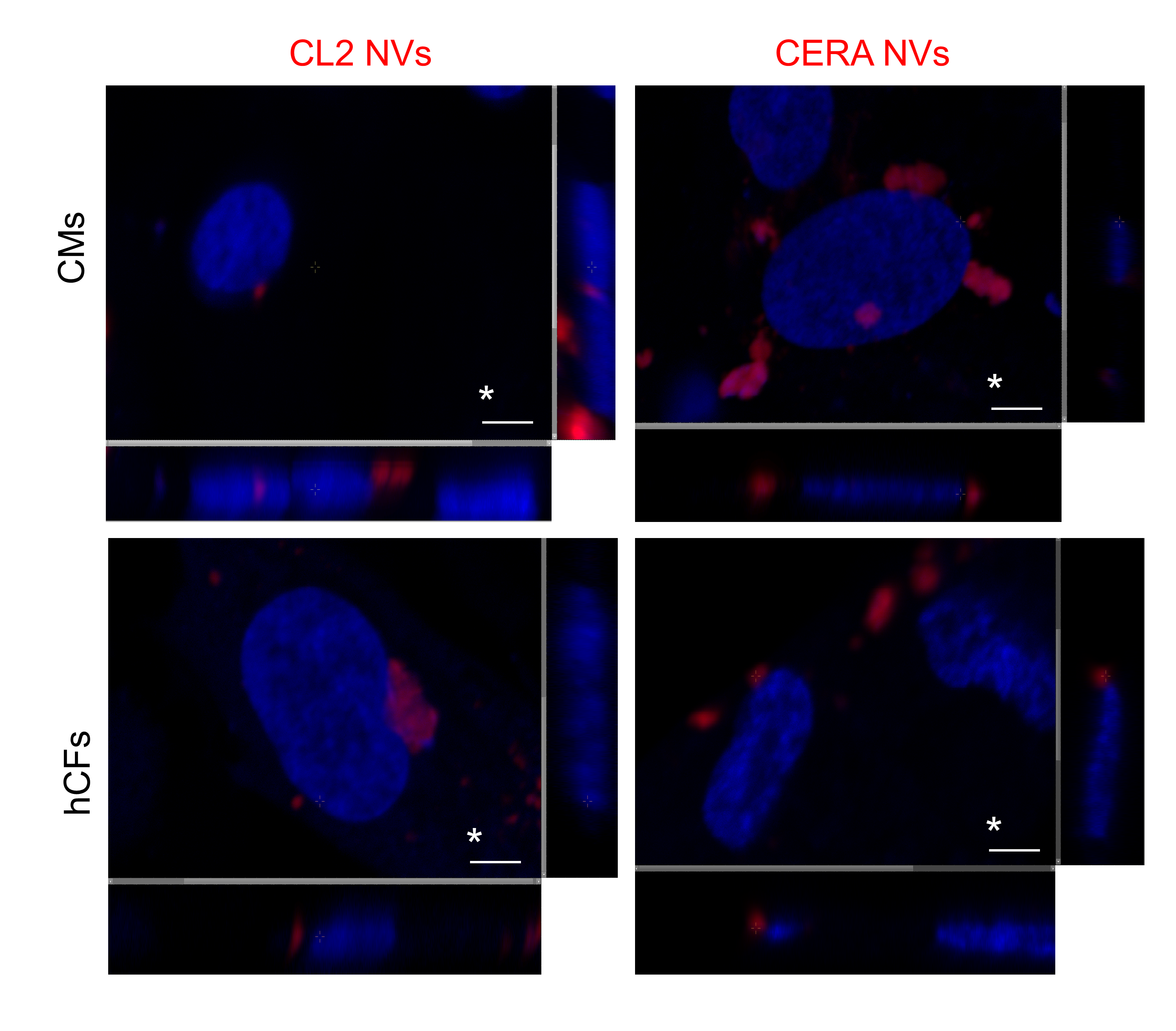

Supplement: Supplementary file 1 [file ijms-23-14334-s001.zip › Supplementary Figure S6.png]

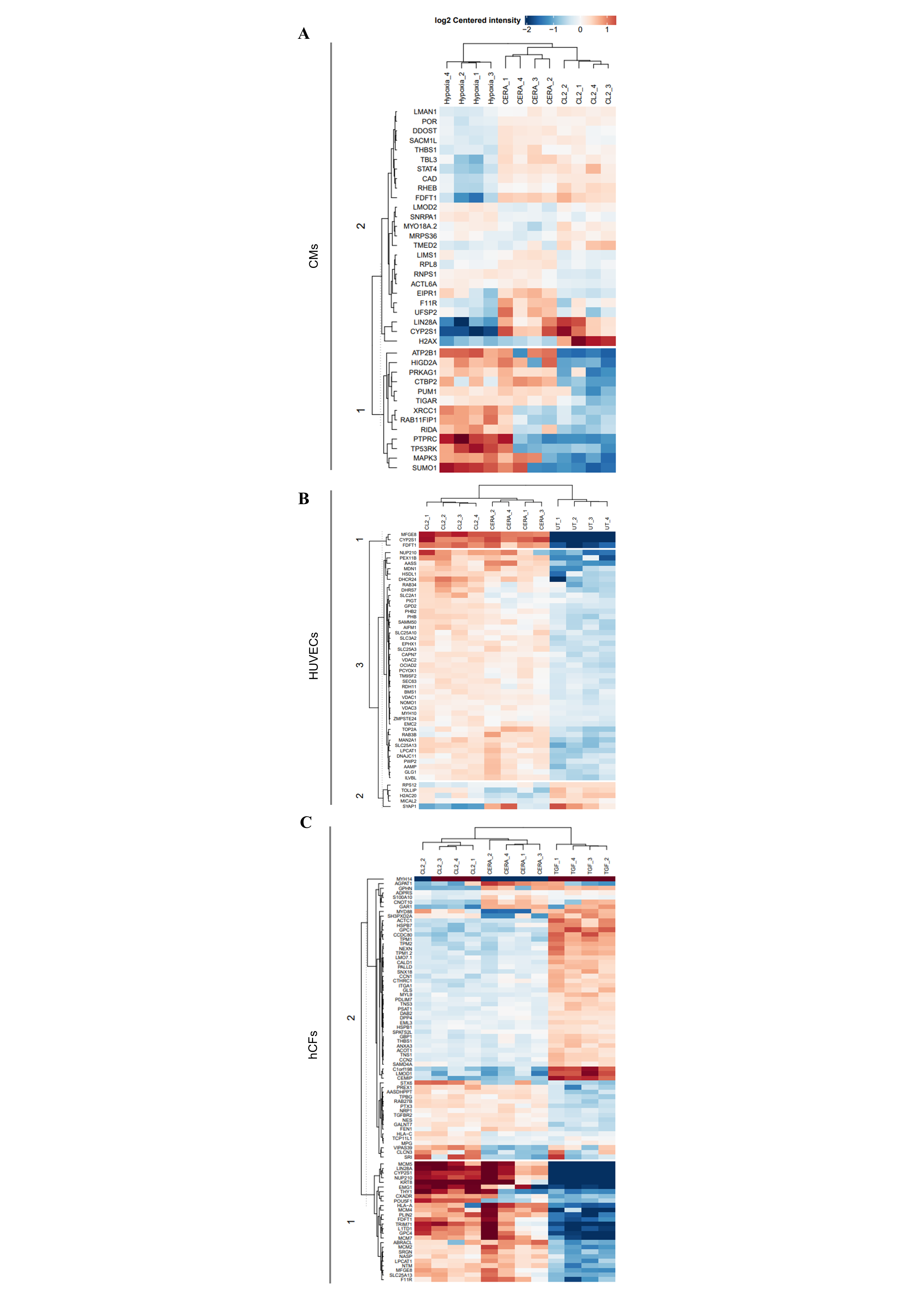

Supplement: Supplementary file 1 [file ijms-23-14334-s001.zip › Supplementary Figure S7.png]
